# Correlation between ^18^F-1-amino-3-fluorocyclobutane-1-carboxylic acid (^18^F-fluciclovine) uptake and expression of alanine-serine-cysteine-transporter 2 (ASCT2) and L-type amino acid transporter 1 (LAT1) in primary prostate cancer

**DOI:** 10.1186/s13550-019-0518-5

**Published:** 2019-05-31

**Authors:** Irena Saarinen, Ivan Jambor, Mai Kim, Anna Kuisma, Jukka Kemppainen, Harri Merisaari, Olli Eskola, Anna-Riina Koskenniemi, Ileana Montoya Perez, Peter Boström, Pekka Taimen, Heikki Minn

**Affiliations:** 1 Institute of Biomedicine, University of Turku and Department of Pathology, Turku University Hospital, Turku, Finland; 2 Department of Radiology, University of Turku , Turku, Finland; 3 Department of Radiology, Icahn School of Medicine at Mount Sinai, New York, New York, USA; 4 Department of Oral and Maxillofacial Surgery. Plastic Surgery, Gunma University Graduate School of Medicine, Maebashi, Japan; 5 Department of Diagnostic Radiology and Nuclear Medicine, Gunma University Graduate School of Medicine, Maebashi, Japan; 6 Turku PET Centre, Turku, Finland; 7 Department of Oncology and Radiotherapy, Turku University Hospital, Kiinamyllynkatu 4-8, P.O. Box 52, FI-20521 Turku, Finland; 8 Department of Clinical Physiology and Nuclear Medicine, Turku University Hospital, Turku, Finland; 9 Department of Future Technologies, University of Turku, Turku, Finland; 10 Department of Urology, Turku University Hospital, Turku, Finland

**Keywords:** ^18^F-fluciclovine, Amino acid transporter, ASCT2, LAT1, Prostate cancer, PET

## Abstract

**Purpose:**

To evaluate the expression of alanine-serine-cysteine-transporter 2 (ASCT2) and L-type amino acid transporter1 (LAT1) in prostate cancer (PCa) and their impact on uptake of ^18^F-1-amino-3-fluorocyclobutane-1-carboxylic acid (^18^F-fluciclovine) which is approved for the detection of recurrent PCa.

**Methods:**

Twenty-five hormone-naïve patients with histologically confirmed PCa underwent PET/CT before prostatectomy. Dynamic imaging was performed immediately after injection of 368 ± 10 MBq of ^18^F-fluciclovine and the uptake in PCa was expressed as SUV_max_ at six sequential 4-min time frames and as tracer distribution volume (*V*_T_) using Logan plots over 0–24 min. The expression of ASCT2 and LAT1 was studied with immunohistochemistry (IHC) on a tissue microarray (TMA) containing three cores per carcinoma lesion. The TMA slides were scored independently by two trained readers based on visual intensity of ASCT2/LAT1 expression on a four-tiered scale. The correlations between ASCT2/LAT1 staining intensity, SUVmax/*V*_T_, and Gleason grade group (GGG) were assessed using Spearman’s rank correlation coefficient (*ρ*).

**Results:**

Forty tumor foci (> 0.5 mm in diameter, max. 3 per patient) were available for TMA. In visual scoring, low, moderate, and high staining intensity of ASCT2 was observed in 4 (10%), 24 (60%), and 12 (30%) tumors, respectively. No tumors showed high LAT1 staining intensity while moderate intensity was found in 10 (25%), 25 (63%) showed low, and the remaining 5 (12%) were negative for staining with LAT1. Tumors with GGG > 2 showed significantly higher uptake of ^18^F-fluciclovine and higher LAT1 staining intensity (*p* < 0.05). The uptake of ^18^F-fluciclovine correlated significantly with LAT1 expression (*ρ* = 0.39, *p* = 0.01, for SUV_max_ at 2 min and *ρ* = 0.39, *p* = 0.01 for *V*_T_). No correlation between ASCT2 expression and ^18^F-fluciclovine uptake or GGG was found.

**Conclusions:**

Our findings suggest that LAT1 is moderately associated with the transport of ^18^F-fluciclovine in local PCa not exposed to hormonal therapy. Both high and low Gleason grade tumors express ASCT2 while LAT1 expression is less conspicuous and may be absent in some low-grade tumors. Our observations may be of importance when using ^18^F-fluciclovine imaging in the planning of focal therapies for PCa.

## Introduction

In spite of an overall excellent prognosis of men diagnosed with localized prostate cancer (PCa), approximately 10–15% of men undergoing surgery or radiotherapy for localized PCa will develop recurrence shown by elevated blood levels of prostate-specific antigen (PSA) [1]. It is imperative to distinguish local recurrence from metastatic disease given the different management of patients with local or advanced relapse. Positron emission tomography (PET) with anti-1-amino-3-[^18^F]-fluorocyclobutane-1-carboxylic acid (^18^F-fluciclovine) is a promising technique for imaging of PCa and since 2016 approved by FDA in the setting of biochemical recurrence [2]. ^18^F-fluciclovine PET seems to be more sensitive than conventional imaging for early detection of recurrence and together with other radiolabeled compounds such as choline or prostate-specific membrane antigen (PSMA) derivatives is likely to promote molecular imaging as the first step in the evaluation of patients presenting with increased PSA after initial treatment [3].

The changing landscape of molecular imaging requires profound study about mechanisms of tracer uptake in PCa where the goal is not only staging but also the selection of targeted therapeutic approaches. ^18^F-fluciclovine is a synthetic L-leucine analog mainly transported to cells by two systems, including sodium-dependent alanine-serine-cysteine transporter 2 (ASCT2) for system ASC and sodium-independent L-type amino acid transporter 1 (LAT1) for system L. [4]. The expression of these amino acid transporters has been shown to be high in cancer cells and the significance of ASCT2 and LAT1 for malignant progression deserved aptly a connotation of “partners in crime” in a seminal review [5]. In particular, high expression of ASCT2 may be linked to a more aggressive biological behavior in PCa and to a poor prognosis in lung and breast cancer [5, 6]. LAT1 supplies amino acids for the proliferation of solid tumors including PCa and head and neck cancer and its important prognostic role in tongue cancer is well demonstrated [7].

A previous report showed that ASCT2 has a major role in ^14^C-fluciclovine uptake into androgen-dependent PCa cells which may parallel the feasibility of ^18^F-fluciclovine to detect PCa before castration resistance [8]. Bearing in mind the relationship of ASCT2 and LAT1 as obligatory amino acid exchangers where leucine and glutamine are driving growth and survival of tumor cells [5], we undertook current study where patients with intermediate to high-risk PCa received ^18^F-fluciclovine PET/CT before robotic prostatectomy. We studied whether expression of ASCT2 and LAT1 correlate with tracer uptake in intraprostatic tumors and with factors determining clinical risk groups of PCa such as serum PSA and Gleason grade.

## Materials and methods

### Patients and study characteristics

A total of 32 men with histologically confirmed intermediate to high-risk PCa scheduled for radical robot-assisted prostatectomy were prospectively enrolled between January 2014 and June 2015 as a part of this prospective registered clinical trial. The ClinicalTrials.gov (https://clinicaltrials.gov/ct2/show/NCT02002455) assigned identifier number is NCT02002455. The study protocol was approved by the Ethics Committee of the Turku PET Centre and Turku University Hospital in Finland. Informed consent was signed by all the patients who participated in the study. The study was conducted in compliance with the current revision of the Declaration of Helsinki guideline. Six patients withdraw from the trial before undergoing PET imaging, and another patient was excluded from the final analysis due to a defect in original tissue processing and unsatisfactory staining at tissue microarray. Thus, the final study cohort included 25 patients. Their mean age and preoperative PSA was 65 years (range 54–75) and 11.9 ng/mL (range, 4.1–35), respectively. The median time from transrectal ultrasound-guided biopsies to PET/CT imaging was 72 days (range, 33–161).

### ^18^F-fluciclovine PET/CT imaging

A combined PET/computed tomography (CT) scanner (DiscoveryTM690, General ElectricMedical Systems) with 64-slice acquisition properties and 3D mode was used. Production of ^18^F-fluciclovine was performed by using FASTlabTM Synthesiser (GE Healthcare). After a minimum of 4-h fast, the patients were placed supine on scanner couch and an ^18^F-fluciclovine tracer (368 ± 10 MBq) was injected into the antecubital vein. Preceded by pre-injection low-dose transmission CT, list-mode acquisition of prostate in the field-of-view was started immediately after injection over the following 20 min; finally, two static bed positions covering prostate and pelvis (4 min each) were acquired. The dynamic data were then reconstructed to five 4 min frames. No adverse events were associated with ^18^F-fluciclovine injections, and all patients tolerated the imaging procedure well. Additionally, patients underwent PET/MRI [9] immediately after PET/CT. Finally, within 1 week of PET/CT imaging, multiparametric MRI (mpMRI) was carried out as previously described [10].

All quantitative corrections were applied to the PET sinogram data to take into account detector dead time, radioactivity decay, random scatter, and photon attenuation. PET images were reconstructed in a 128 × 128 matrix with a voxel size of 5.47 × 5.47 × 3.27 mm^3^, using the VUE Point FX algorithm with time-of-flight technology and a 6-mm Gaussian post-filter and no resolution modeling.

### ^18^F-fluciclovine PET/CT analysis

PET/CT images were co-registered [11] with PET/MRI and mpMRI. We first segmented tracer uptake within prostate covering the whole organ. Within this segmented volume focal intraprostatic regions showing increased tracer uptake were identified and correlated with HE-stained macroslides from radical prostatectomy to select regions of interest (ROI) representing the tumor lesions. These ROIs were analyzed quantitatively by calculating the maximum standardized uptake value of ROI voxel (SUV_max_) over each time frame (2, 6, 10, 14, 18, 22 min) according to the following formula: SUV = [tissue radioactivity concentration (Bq/ml) × body weight(g)]/injected dose(Bq). The data sets were analyzed using Advantage Workstation (version4.4, General Electric Medical Systems, Milwaukee, WI, USA).

In addition, Logan plots [12] with a reference region in the iliac/femoral artery were performed to estimate the tracer distribution volume (*V*_T_) based on the assumption that transport of ^18^F-fluciclovine into cells is similar to reversible receptor binding kinetics.

### Histological analysis

Robot-assisted radical prostatectomy was performed within 4 weeks after the hybrid PET studies. The prostate specimens were fixed in 10% buffered formalin for 24–48 h. Thereafter, the prostate surfaces (left, right, and anterior) were inked with different colors to preserve the orientation of the prostate gland and to allow correlation with PET/CT. Whole-mount axial macro-sections were obtained at 5–6 mm intervals transversely in a plane perpendicular to the long axis of the prostate gland in superior-inferior direction, similar to axial images of PET/CT. For easier evaluation of the capsular status of the inferior region, the most apical macro-section tissue block was further sectioned in coronal orientation. In contrast, the first transversal section at the base was further sectioned in sagittal orientation for easier evaluation of the seminal vesicle invasion [13, 14]. Four micrometers of whole-mount sections from each macroblock was cut and stained with hematoxylin and eosin. Each individual tumor focus was graded separately based on the International Society of Urological Pathology (ISUP) guidelines [15]: group 1—GS ≤ 3 + 3, group 2—GS 3 + 4, group 3—GS 4 + 3, group 4—GS 4 + 4/3 + 5/5 + 3, group 5—GS 4 + 5/5 + 4/5 + 5. Any tertiary Gleason pattern representing less than 5% of tumor volume was taken into account in grading and marked as +. Only tumor foci > 0.5 cm in diameter, as defined using whole mount prostatectomy sections, were included in the analysis. In total, 423 lymph nodes were removed from 25 patients. Twenty-three (5%, 23/423) of them harbored metastases in seven (28%) patients.

### Tissue microarray (TMA)

Tissue microarrays (TMA) were constructed of formalin fixed paraffin embedded prostatic tissue material. Three adjacent tissue cores of 1 mm in diameter were punched from each patient’s index tumor and one core from morphologically benign tissue from the same paraffin block. Whenever present, additional three cores were punched from separate secondary/tertiary carcinoma lesion (*n* = 15) and from prostatic intraepithelial neoplasia (PIN) lesions (*n* = 4). Furthermore, two 1 mm tissue cores were punched from metastatic lymph nodes with sufficient material (*n* = 3). The tissue cores from the donor blocks were transferred to the recipient TMA block containing control tissue samples from the placenta, liver, pancreatic adenocarcinoma, and mammary ductal adenocarcinoma.

### Immunohistochemistry (IHC)

TMA tissue sections were cut at 4 μm, paraffin was removed with xylene and the sections were rehydrated with graded series of alcohol. ASCT2 staining was carried out using BenchMark XT automated IHC/ISH slide staining system (Ventana Medical Systems, Inc.), Cell Conditioning Solution (CC1) as a pretreatment and rabbit polyclonal ASCT2-N primary antibody (1:300, kindly provided by prof. Y. Kanai, Osaka University, Japan) with 32 min incubation time. For LAT1, epitope unmasking was done by microwaving the slides in Tris-HCl buffer (pH 9) 2 × 7 min and the staining was carried out with Lab Vision autostainer (Thermo Fisher Scientific) using rabbit polyclonal LAT1 primary antibody (1:750, prof. Y. Kanai, Osaka University, Japan) for 60 min. Primary LAT1 antibodies were detected with PowerVision Poly-HRP anti-mouse/anti-rabbit IHC system (Leica BioSystems). Finally, the slides were also counterstained with hematoxylin.

The immunohistochemical stainings were evaluated independently by two trained readers blinded to clinical, imaging data as well as Gleason scoring. The evaluation was done according to the visual intensity of carcinoma cells as follows: score 0 (negative staining), 1 (low intensity), 2 (moderate intensity), and 3 (high intensity). Whenever the original evaluations of readers were not convergent, the specific spots were re-evaluated by both readers to find consensus. For each PCa lesion, the average staining intensity of all evaluated tumor cores was used for statistical analysis.

### Statistical analysis

Normally distributed continuous variables are given as means and standard deviations, variables not following normality as medians and interquartile ranges, and categorical variables as frequencies and proportions. The Kolmogorov–Smirnov test was used to check normality. ANOVA with the Bonferroni test or Kruskal–Wallis test with Dunn’s test or Mann-Whitney *U* test or unpaired *t* test with Welch’s correction were used to compare parameter values for different tissue/cancer types, when appropriate. The correlations between ASCT2/LAT1 staining intensity and SUV and PSA values were assessed using Spearman’s rank correlation coefficient. Two-sided *p* values were calculated. A *p* < 0.05 was considered statistically significant. Statistical analyses were performed using MATLAB (Mathworks Inc., Natick, MA) and/or GraphPad Prism, version 5.00 (GraphPad Software, San Diego, CA). Post-processing codes as well as all imaging acquisition protocols are freely available upon request.

## Results

### Tissue microarray

In total, 50 tumor foci were identified in whole-mount prostatectomy samples of the included 25 patients. Forty (80%, 40/50) of those were > 0.5 cm in diameter and eligible for comparison between tracer uptake and amino acid transporter expression. Tissue microarray was constructed and successfully analyzed from all these 40 tumor foci (maximum 3 per patient), of which 9, 11, 10, 0, and 10 represented Gleason grade groups 1, 2, 3, 4, and 5, respectively (Table 1).

**Table 1:** Histological characteristics and findings at immunohistochemistry evaluating amino acid transporter expression of 40 tumor foci in 25 patients with localized prostate cancer. Note that no tumors were assigned in Gleason grade group 4

Gleason grade/Gleason grade group	Number	Percent
3 + 3/1	9	22.5
3 + 4/2	11	27.5
4 + 3/3	10	25.0
4 + 5/5	8	20
5 + 4/5	2	5
ASCT2 staining intensity		
Low (1)	4	10
Moderate (2)	24	60
High (3)	12	30
LAT1 staining intensity		
Negative (0)	5	12
Low (1)	25	63
Moderate (2)	10	25

### Dynamic ^18^F-fluciclovine PET imaging

All 40 analyzed tumors showed variable but increased uptake of ^18^F-fluciclovine compared to normal prostate tissue. The uptake in PCa peaked early between 4 to 8 min, and the median (interquartile range) SUV_max_ of the 40 tumors over the five dynamic and one static frame was 3.7 (2.5–5.7), 5.4 (3.9–7.1), 4.6 (3.9–7.1), 4.3 (3.7–5.9), 4.0 (3.4–4.9), and 3.0 (2.6–3.6) at 2, 6, 10, 14, 18, and 22 min. The volume of distribution of tracer expressed as *V*_T_ was 2.5 (2.0–3.7).

### Immunohistochemical findings

**Fig. 1:**
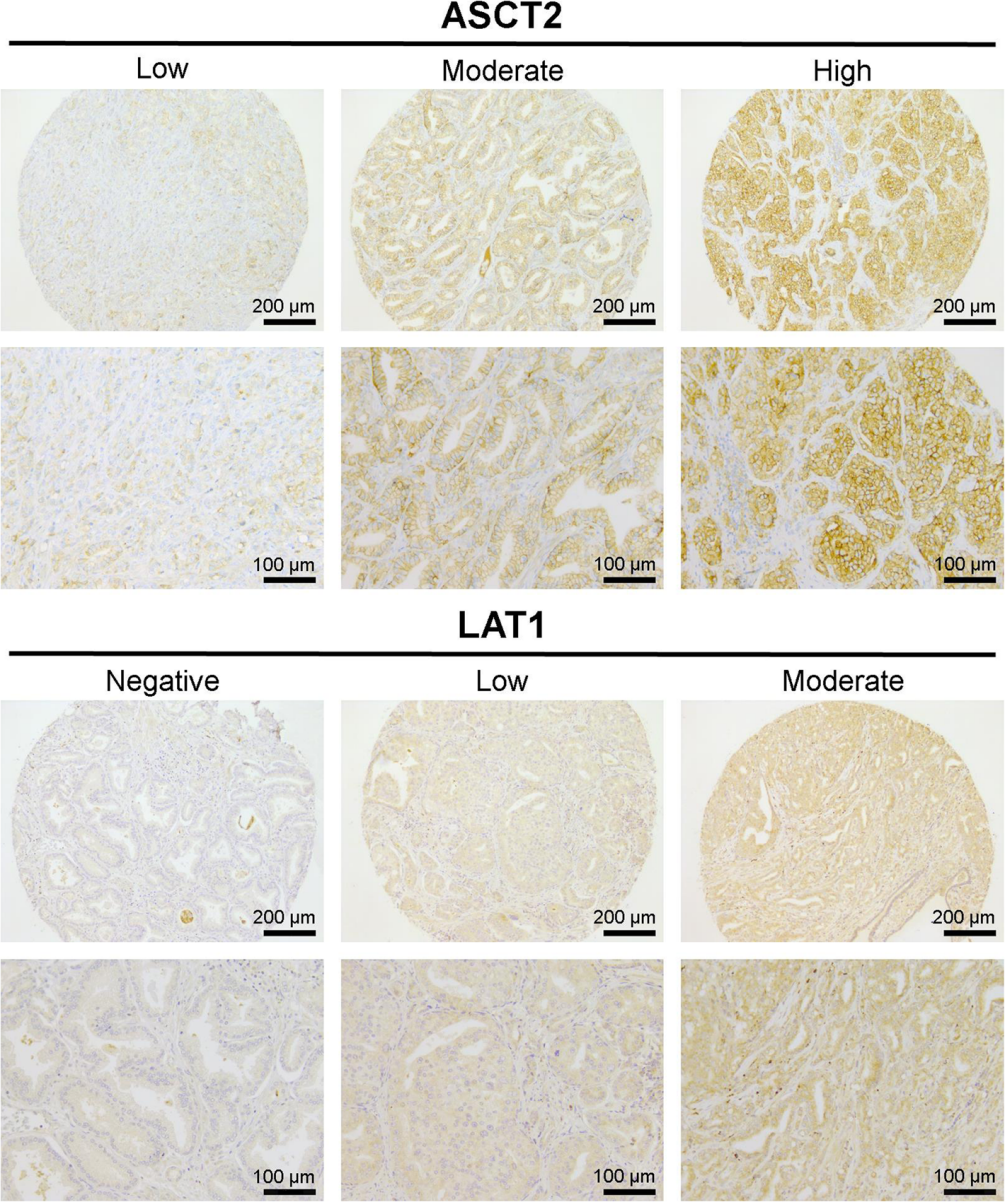
Representative examples of different immunohistochemical (IHC) staining intensities of ASCT2 and LAT1 in tissue microarray (TMA). Low, moderate, and high IHC staining intensities (score 1–3) for ASCT2 and negative, low, and moderate IHC staining intensities (score 0–2) for LAT1 are shown at low (× 10, scale bar 200 μm) and high (× 20, scale bar 100 μm) magnification

ASCT2 antibody stained predominantly the plasma membrane of epithelial cells in both benign and malignant prostatic glands, as well as in prostatic intraepithelial neoplasia (PIN) lesions, while LAT1 staining was mostly cytoplasmic (Fig. 1). ASCT2 staining intensity in morphologically benign glands varied from weak to high (score 1–3) in basal glandular cells while luminal cells had generally lower ASCT2 staining intensity (score 0–2). LAT-1 staining, on the other hand, was virtually negative (score 0) in normal basal cells and weak in 83% of normal luminal cells. Twelve out of 40 tumors (30%) demonstrated high ASCT2 staining intensity while 24 (60%) showed moderate and 4 (10%) low intensity, respectively. LAT1 intensity, in turn, was scored as moderate in 10 (25%) tumors and from the remaining tumors 25 (63%) showed low and 5 (12%) no staining with LAT1 in all three cores (Table 2). Of note is that no tumor had high LAT 1 staining intensity and there was no correlation between the staining intensities of the two amino acid transporters (*ρ* = − 0.02, *p* = 0.90). One out of three metastatic lymph nodes showed high ASCT2 intensity and low LAT1 intensity while two other nodes both showed low ASCT2 and LAT1 intensity.

**Table 2:** Distribution of ASCT2 and LAT1 scores in 40 prostate cancer tumors

	ASCT2 score 1	ASCT2 score 2	ASCT2 score3
LAT1 score 0	0	4	1
LAT1 score 1	4	13	8
LAT1 score 2	0	7	3

### Correlation of ^18^F-fluciclovine uptake with Gleason grade groups and expression of amino acid transporters

**Fig. 2:**
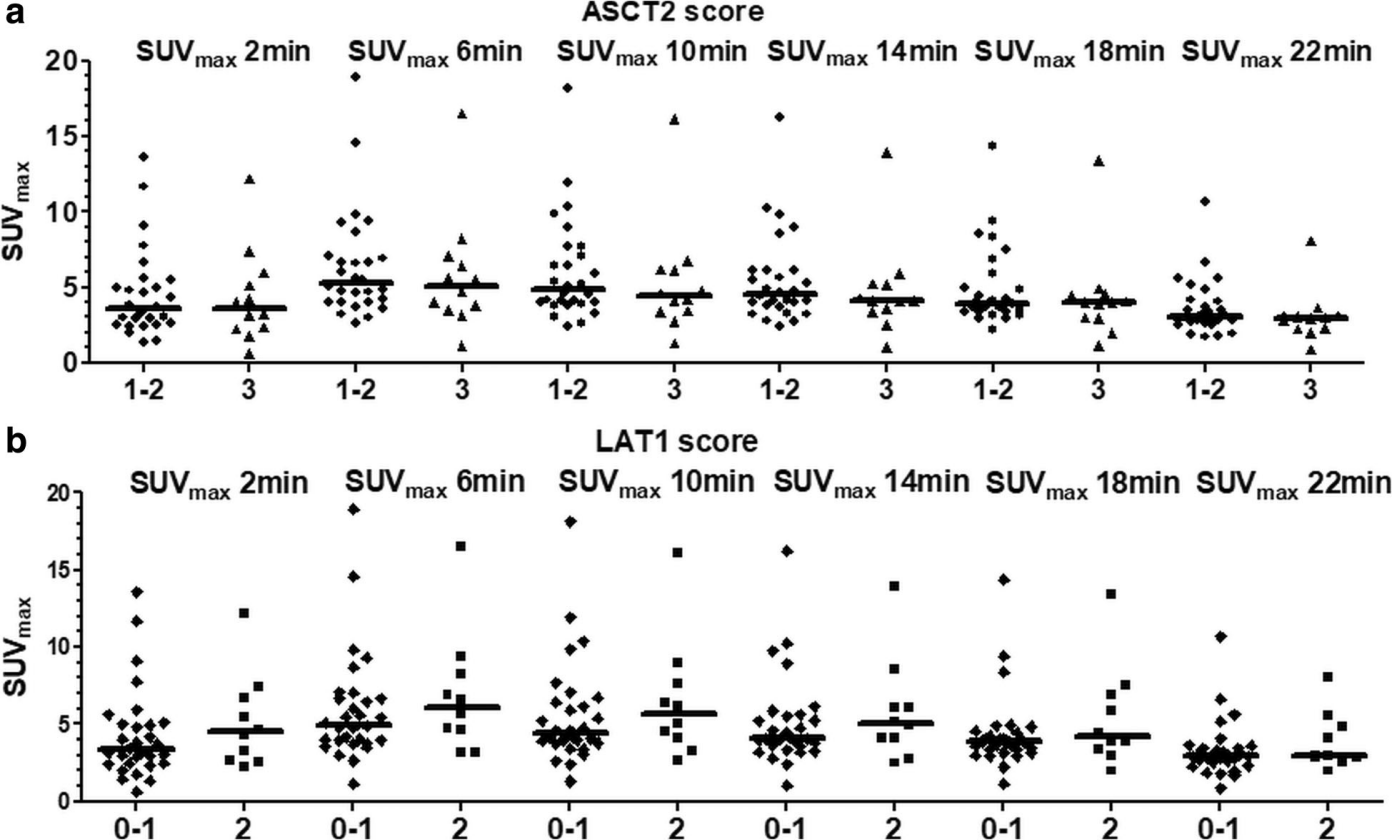
Relationship between uptake of ^18^F-fluciclovine and amino acid transporter expression in localized prostate cancer. Time course of uptake, expressed as SUV_max_ of six consecutive 4-min frames and indicated by their middle time point is shown in comparison to ASCT2 (**a**) and LAT1 (**b**) represented in two staining intensity subgroups. For ASCT2, 1–2 denotes low-to-moderate and 3 high expression in **a**. For LAT1, 0–1 denotes negative-to-low and 2 moderate expression in **b**, respectively

**Fig. 3:**
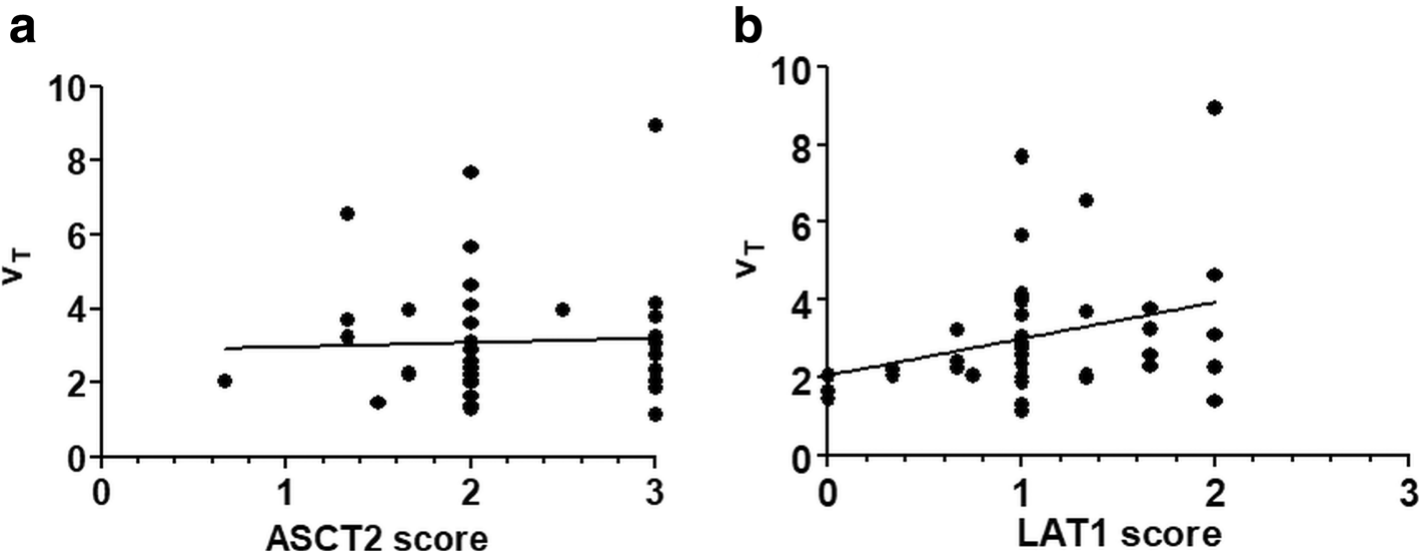
Correlation between ^18^F-fluciclovine distribution volume (*V*_T_) and amino acid transporter expression. On the *x*-axis, the average staining intensities of ASCT2 and LAT1 in each separate tumor lesion are depicted. Linear regression is shown to demostrate correlation between V_T_ and ASCT2/LAT1 scores

Figure 2 shows the progression of tracer uptake in succession over the six 4 min frames against amino acid transporter expression divided into two groups based on staining intensity. Although the intensity of staining with ASCT2 was in general high or moderate, there was no correlation between expression of ASCT2 with SUV_max_ at any of the six frames (Fig. 2a). In contrast, LAT1 and SUV_max_ showed significant correlation over the first four frames which declined and became insignificant towards the end of the acquisition (Fig. 2b). The Spearman’s rank correlation coefficients (*ρ*) of the individual frames for SUV_max_ vs. LAT1 were 0.39 (*p* = 0.01), 0.34 (*p* = 0.03), 0.36 (*p* = 0.03), 0.34 (*p* = 0.03), 0.31 (*p* = 0.05), and 0.24 (*p* = 0.13), respectively. Similar to SUV_max_, the correlation between *V*_T_ and LAT1 staining intensity was statistically significant at *ρ* = 0.34 and *p* = 0.04 while no significant association between graphical analysis expressed as *V*_T_ and ASCT2 was seen (Fig. 3).

**Fig. 4:**
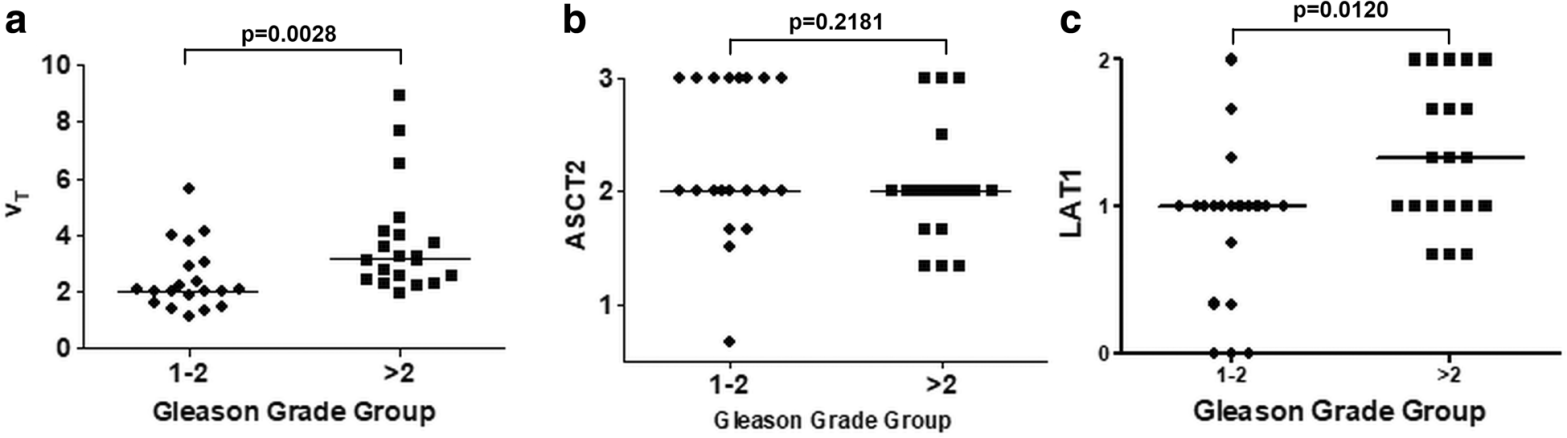
Correlation between ^18^F-fluciclovine distribution volume (*V*_T_), amino acid transporter expression (ASCT2/LAT1 staining intensities), and Gleason grade groups (GGG). On the *x*-axis, GGG ≤ 2 and GGG > 2 are shown separately *P*-values are shown for the differences between GGG ≤ 2 and GGG > 2

*V*_T_ and LAT1 staining intensities (negative-to-low vs. moderate) of lesions with GGG higher than 2 were significantly higher than those of lesions with GGG 2 or lower (Fig. 4; *p* = 0.003 and 0.01 for *V*_T_ and LAT1, respectively). In contrast, the high intensity of ASCT staining was commonly seen independent of GGG (Fig. 4). As expected, there was no correlation between uptake of ^18^F-fluciclovine expressed as SUV_max_, or *V*_T_ and serum PSA. Similarly, ASCT2 or LAT1 staining intensities did not correlate with serum PSA.

**Fig. 5:**
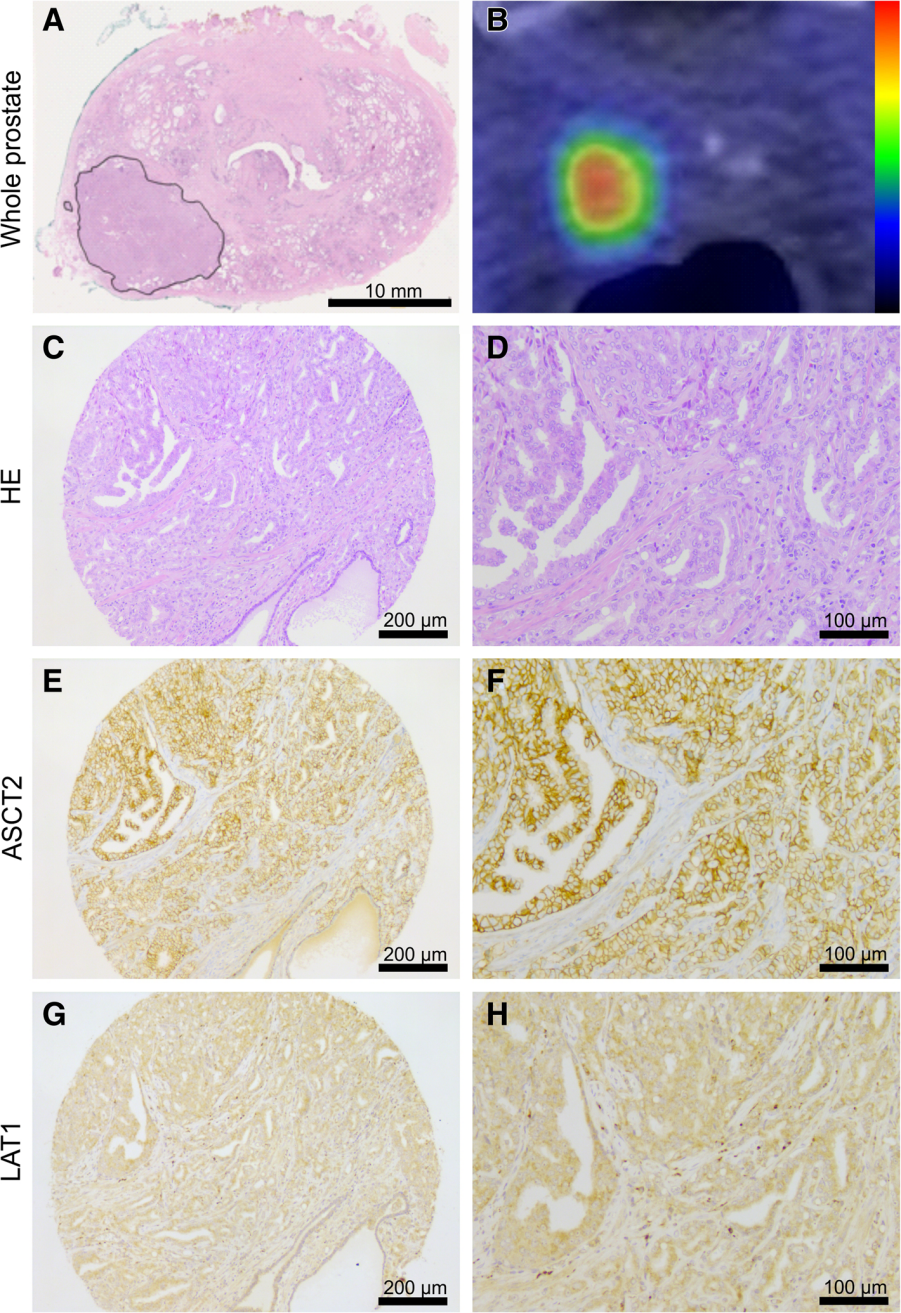
A representative case of a 66-year-old patient with preoperative PSA of 4.6 ng/ml. HE staining of the whole mount prostatectomy section indicates a solitary lesion in the right peripheral lobe (**a**). Axial PET/CT shows conspicuous high ^18^F-fluciclovine uptake (SUV_max_ at 22 min = 9.5) in the tumor (**b**). Low (× 10, scale bar 200 μm) and high magnification (× 20, scale bar 100 μm) images of HE-stained TMA core from the tumor show Gleason 4 + 5 adenocarcinoma (**c**, **d**). Parallel sections used for immunohistochemistry show high ASCT2 (**e**, **f**) and moderate LAT1 (**g**, **h**) staining intensity

**Fig. 6:**
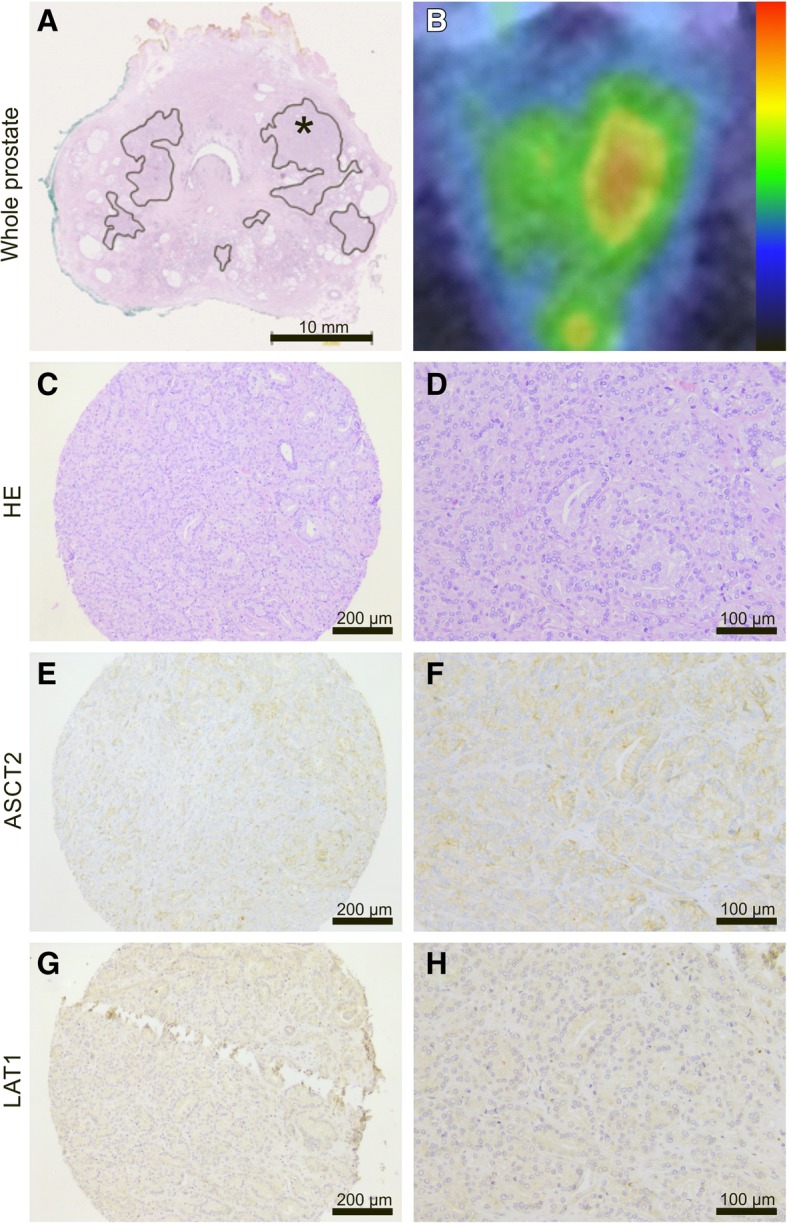
A representative case of a 67-year-old patient with preoperative PSA of 6.2 ng/ml. HE staining of whole-mount prostatectomy section indicates a bilobar adenocarcinoma with index lesion in the left lobe (marked with *) and another tumor in the right lobe (**a**). Axial PET/CT shows moderate ^18^F-fluciclovine uptake in the index lesion (SUV_max_ at 22 min = 3.2) and low uptake in the right lobe tumor (**b**). Low (× 10) and high (× 20) magnification images of HE-stained TMA core from the left index tumor shows Gleason 4 + 5 adenocarcinoma (**c**, **d**). Parallel sections show low ASCT2 (**e**, **f**) and LAT1 (**g**, **h**) staining intensity

Representative cases with differential staining intensity of amino acid transporters are presented in Figs. 5 and 6. A patient in the high clinical risk group having a lesion with GS 4 + 5 = 9 (GGG 5) PCa with high (SUV_max_ 8.1) ^18^F-fluciclovine index tumor uptake demonstrated high ASCT2 and moderate LAT1 intensity (Fig. 5). Another patient (Fig. 6) with an index tumor GS 4 + 5 = 9 (GGG 5) lesion demonstrated low ^18^F-fluciclovine uptake of both left index (SUV_max_ 3.2) and contralateral right lobe tumor where staining intensities were low-to-moderate for ASCT2 and low for LAT1.

## Discussion

We studied the relationship of sodium-dependent and sodium-independent amino acid transporters ASCT2 and LAT1 with ^18^F-fluciclovine uptake in primary prostate cancer patients who underwent PET/CT before robotic prostatectomy. We found that expression of ASCT2 did not correlate with ^18^F-fluciclovine uptake and did not differ significantly between tumors with different GGG. In contrast, expression of LAT1 was significantly increased in tumors with higher GGG and in those with higher uptake of ^18^F-fluciclovine which may reflect the characteristic of the tracer as a leucine analog and its association with a higher GGG. LAT1 expression was generally lower than that of ASCT2 but no correlation was found between LAT1 and ASCT2 staining intensities.

Glutamine is the most abundant amino acid in plasma and after transport via ASCT2 acts as an alternative source for citric acid cycle in tumor cells where it may be favored for oxidation [16]. ASCT2 is regulated by androgen receptor (AR) [17], and a previous study has demonstrated that patients undergoing neoadjuvant hormonal therapy showed decreased ASCT2 protein expression [16]. The preference for glutamine as a substitute for glucose in the respiratory cycle might contribute to the typically low 2-deoxy-[^18^F]fluoro-D-glucose uptake in early PCa. Second, both glutamine and leucine are important sensors fueling mechanistic target of rapamycin complex 1 (mTORC1) activity, which drives neoplastic growth and protein translation [18, 19]. Thus, successful pharmacological inhibition of AR and mTORC1 pathway may result in decreased ^18^F-fluciclovine signal, and PET with ^18^F-fluciclovine might be beneficial for follow-up of patients with hormonal therapy or drugs affecting these pathways [16]. How switch of metabolism to higher dependence on LAT1 compared to ASCT2 would modify ^18^F-fluciclovine uptake after acquired androgen resistance and tumor progression remains thus far open. Although we did not find the correlation between ASCT2 and ^18^F- fluciclovine uptake (SUV_max_ and *V*_T_), ^18^F-fluciclovine-positive primary PCa is likely to depend on ASCT2 as well to maintain growth stimulated by androgen signaling. Since ASCT2 is the major glutamine transporter [5], ^18^F-fluciclovine may in fact serve as a surrogate marker of increased glutamine metabolism.

Li et al. suggested that ASCT2 expression in PCa correlates with GS and that the expression appears to be related to tumor aggressiveness and poor survival [20]. However, they also found high-level expression in the cytoplasm of normal epithelial cells of the prostate which was significantly higher than that of PCa and benign prostatic hyperplasia (BPH). Similarly to ASCT2, Sakata et al. suggested that LAT1 is a novel independent biomarker for high-grade PCa and shows prognostic significance [21]. In contrast to LAT1 which was higher in GGG > 2 tumors, our study failed to find a statistically significant correlation between ASCT2 expression and GGG. These discrepancies might be due to limited patient number of our study and differences in analytical methods used to assess amino acid transporter expression. Second, high expression of ASCT2 in epithelial cells seen by us and Li et al. [20] may explain false positive findings of ^18^F-fluciclovine imaging in the primary evaluation of PCa and possibly after treatment as well. Nevertheless, the association between LAT1 with both SUV_max/_*V*_T_ and GGG indicates that ^18^F-fluciclovine imaging could assist in evaluating cancer aggressiveness and when co-registered with multiparametric MRI might be helpful in guiding targeted biopsy or focal treatments of multifocal PCa [9].

The low number of lymph node metastases included in our study limits conclusions about amino acid transporter expression in metastatic disease. The differential expression pattern in multiple intraprostatic tumors and nodal metastases might reflect the heterogeneity of PCa, and we hypothesize that shift to higher dependency on sodium-independent transport is more common during disease progression. This would be coupled with a decrease in glutamine use and higher dependence on glucose which is in concert with the fact that aggressive forms of PCa may be ^18^F-FDG positive. Analysis of amino acid and glucose transporter expression in tumors of the same patient over the progression of disease to castration-resistant phase would shed light on this.

In the current study, TMA approach with three cores from each carcinoma lesion was used, and their representativeness in relation to the entire malignant lesion remains obscure. Second, the region of interest representing SUV_max_ does not necessarily match perfectly spatially the respective region of the tissue samples. Third, although two independent readers were used for scoring of ASCT2/LAT1 expression, the procedure is subjective and statistical comparison has to be made with caution. The fourth limitation is that we did not include multiple cores from morphologically benign prostatic tissue or from hyperplastic nodules into TMA analysis. Spatial correlation between expression of ASCT2/LAT1 in these benign tissues and tracer uptake would have been even more challenging than that of PCa foci. Being aware of these limitations, we decided to focus on clarifying the differential role of two amino acid transporters in contributing to ^18^F-fluciclovine uptake in PCa.

Our observations do not fully correspond with pre-clinical data on amino acid transport where cell lines with and without androgen dependency have been evaluated [4, 8, 22]. The copious expression of ASCT2 and LAT1 in the majority of human cancers and several cancer cell lines is in line with the general success of amino acid PET in oncology. We feel that understanding the specific role of individual transport mechanisms is important for a clinical application since they may vary in tumors over disease stage and phase. [^18^F]-fluciclovine as an unmetabolized leucine analog appears to suit well in the study of amino acid metabolism. In pre-clinical studies, it was shown that [^18^F]- fluciclovine is transported by both ASCT2 and LAT1 (23) and thus reflects the balance between intracellular glutamine and leucine which are reciprocally exchanged to maintain the intracellular amino acid pool for growth and survival [5].

In conclusion, we have found that the increased uptake of ^18^F- fluciclovine seen in intraprostatic tumors is in 90% of the tumor (35/40) associated with moderate or high ASCT2 expression. However, our findings failed to support pre-clinical observations about the essential contribution of sodium-dependent ASCT2 transport system in the uptake of ^18^F-fluciclovine in local PCa. On the other hand, we have found a significant association of [^18^F]-fluciclovine uptake with LAT1 staining intensity and GGG. Furthermore, aggressive tumors with higher GGG > 2 showed higher LAT1 staining intensity compared to those having GGG ≤ 2. The association of amino acid transporters with metabolic pathways of glutamine and leucine highlight the potential of ^18^F-fluciclovine to monitor metabolic changes in PCa over the course of the disease. These changes are likely to depend on androgen sensitivity and clonal evolution leading to new genomic alterations and finally castration resistance.

## Abbreviations

ASCT2: Sodium dependent alanine-serine-cysteine transporter 2
GGG: Gleason grade group
GS: Gleason score
LAT1: Sodium independent L-type amino acid transporter 1
PCa: Prostate cancer
PET: Positron emission tomography
PIN: Prostatic intraepithelial neoplasia
PSA: Prostate-specific antigen
ROI: Regions of interest
SUV_max_: Maximum standardized uptake value
TMA: Tissue microarray
*V*_T_: Tracer distribution volume

## References

[1] Hamdy FC, Donovan JL, Lane JA, Mason M, Metcalfe C, Holding P (2016). 10-year outcomes after monitoring, surgery, or radiotherapy for localized prostate cancer. N Engl J Med.

[2] Savir-Baruch B, Zanoni L, Schuster DM (2017). Imaging of prostate cancer using fluciclovine. PET Clin.

[3] Wibmer AG, Burger IA, Sala E, Hricak H, Weber WA, Vargas HA (2016). Molecular imaging of prostate cancer. Radiographics.

[4] Okudaira H, Shikano N, Nishii R, Miyagi T, Yoshimoto M, Kobayashi M (2011). Putative transport mechanism and intracellular fate of trans-1-amino-3-18F-fluorocyclobutanecarboxylic acid in human prostate cancer. J Nucl Med.

[5] Fuchs BC, Bode BP (2005). Amino acid transporters ASCT2 and LAT1 in cancer: partners in crime?. Semin Cancer Biol.

[6] Shimizu K, Kaira K, Tomizawa Y, Sunaga N, Kawashima O, Oriuchi N (2014). ASC amino-acid transporter 2 (ASCT2) as a novel prognostic marker in non-small cell lung cancer. Br J Cancer.

[7] Toyoda M, Kaira K, Ohshima Y, Ishioka NS, Shino M, Sakakura K (2014). Prognostic significance of amino-acid transporter expression (LAT1, ASCT2, and xCT) in surgically resected tongue cancer. Br J Cancer.

[8] Ono M, Oka S, Okudaira H, Nakanishi T, Mizokami A, Kobayashi M (2015). [(14)C]Fluciclovine (alias anti-[(14)C]FACBC) uptake and ASCT2 expression in castration-resistant prostate cancer cells. Nucl Med Biol.

[9] Jambor I, Kuisma A, Kähkönen E, Kemppainen J, Merisaari H, Eskola O (2018). Prospective evaluation of 18F-FACBC PET/CT and PET/MRI versus multiparametric MRI in intermediate- to high-risk prostate cancer patients (FLUCIPRO trial). Eur J Nucl Med Mol Imaging.

[10] Jambor I, Kähkönen E, Taimen P, Merisaari H, Saunavaara J, Alanen K (2015). Prebiopsy multiparametric 3T prostate MRI in patients with elevated PSA, normal digital rectal examination, and no previous biopsy. J Magn Reson Imaging.

[11] Jambor I, Borra R, Kemppainen J, Lepomäki V, Parkkola R, Dean K (2012). Improved detection of localized prostate cancer using co-registered MRI and 11C-acetate PET/CT. Eur J Radiol.

[12] Logan J (2000). Graphical analysis of PET data applied to reversible and irreversible tracers. Nucl Med Biol.

[13] Jambor I, Merisaari H, Taimen P, Boström P, Minn H, Pesola M (2015). Evaluation of different mathematical models for diffusion-weighted imaging of normal prostate and prostate cancer using high b-values: a repeatability study. Magn Reson Med.

[14] Jambor I, Borra R, Kemppainen J, Lepomäki V, Parkkola R, Dean K (2010). Functional imaging of localized prostate cancer aggressiveness using 11C-acetate PET/CT and 1H-MR spectroscopy. J Nucl Med.

[15] Epstein JI, Egevad L, Amin MB, Delahunt B, Srigley JR, Humphrey PA (2016). The 2014 International Society of Urological Pathology (ISUP) Consensus Conference on Gleason Grading of Prostatic Carcinoma: Definition of Grading Patterns and Proposal for a New Grading System. Am J Surg Pathol.

[16] Wang Q, Hardie R, Hoy AJ, van Geldermalsen M, Gao D, Fazli L (2015). Targeting ASCT2-mediated glutamine uptake blocks prostate cancer growth and tumour development. J Pathol.

[17] Wang Q, Tiffen J, Bailey CG, Lehman ML, Ritchie W, Fazli L (2013). Targeting amino acid transport in metastatic castration-resistant prostate cancer: effects on cell cycle, cell growth, and tumor development. J Natl Cancer Inst.

[18] van Geldermalsen M, Wang Q, Nagarajah R, Marshall AD, Thoeng A, Gao D (2016). ASCT2/SLC1A5 controls glutamine uptake and tumour growth in triple-negative basal-like breast cancer. Oncogene.

[19] Goberdhan DCI, Wilson C, Harris AL (2016). Amino acid sensing by mTORC1: intracellular transporters mark the spot. Cell Metab.

[20] Li R, Younes M, Frolov A, Wheeler TM, Scardino P, Ohori M (2003). Expression of neutral amino acid transporter ASCT2 in human prostate. Anticancer Res.

[21] Sakata T, Ferdous G, Tsuruta T, Satoh T, Baba S, Muto T (2009). L-type amino-acid transporter 1 as a novel biomarker for high-grade malignancy in prostate cancer. Pathol Int.

[22] Oka S, Okudaira H, Yoshida Y, Schuster DM, Goodman MM, Shirakami Y (2012). Transport mechanisms of trans-1-amino-3-fluoro[1-(14)C]cyclobutanecarboxylic acid in prostate cancer cells. Nucl Med Biol.

